# Decision to Delivery Interval, Perinatal Outcome and Factors Following Emergency Cesarean Section in Southern Ethiopia

**DOI:** 10.4314/ejhs.v33i1.6

**Published:** 2023-01

**Authors:** Kassaw Beyene, Kassahun Fekadu, Manaye Yihune, Yosef Alemayehu, Dagninet Alelign, Gedife Ashebir, Biresaw Wassihun, Abrham Debeb

**Affiliations:** 1 Department of Midwifery, College of Medicine and Health Sciences, Arba Minch University, Arba Minch, Ethiopia; 2 School of Public Health, College of Medicine and Health Sciences, Arba Minch University, Arba Minch, Ethiopia; 3 Departement of Medical laboratory, College of Medicine and Health Sciences, Arba Minch University, Arba Minch, Ethiopia; 4 Department of Midwifery, College of Health Sciences, Injibara University, Injibara, Ethiopia; 5 Department of Midwifery, College of Medicine and Health Sciences, Wachamo University, Hossana , Ethiopia

**Keywords:** Decision to delivery interval, perinatal outcomes, Emergency cesarean section, Southern Ethiopia

## Abstract

**Background:**

The interval between the decision for an emergency cesarean section and the delivery of the fetus should be made within 30 minutes. In a setting like Ethiopia, the recommendation of 30 minutes is unrealistic. Decision to delivery interval should, therefore, be considered as vital in improving perinatal outcomes. This study aimed to assess the decision to delivery interval, its perinatal outcomes, and associated factors.

**Methods:**

A facility-based cross-sectional study was employed, and a consecutive sampling technique was used. Both the questionnaire and the data extraction sheet were used, and data analysis was done using a statistical package for social science version 25 software. Binary logistic regression was used to assess the factors associated with decision to delivery interval. P-value < 0.05 level of significance with a 95% Confidence interval was considered statistically significant.

**Results:**

Decision-to-delivery interval below 30 minutes was observed in 21.3% of emergency cesarean sections. Category one (AOR=8.45, 95% CI, 4.66, 15.35), the presence of additional OR table (AOR=3.31, 95% CI, 1.42, 7.70), availability of materials and drugs (AOR=4.08, 95% CI, 1.3, 12.62) and night time (AOR=3.08, 95% CI, 1.04, 9.07) were factors significantly associated. The finding revealed that there was no statistically significant association between prolonged decisions to delivery interval with adverse perinatal outcomes.

**Conclusions:**

Decision-to-delivery intervals were not achieved within the recommended time interval. The prolonged decision to delivery interval and adverse perinatal outcomes had no significant association. Providers and facilities should be better equipped in advance and ready for a rapid emergency cesarean section.

## Introduction

The provision of quality obstetric care needs timely access to emergency cesarean section (ECS). This, in turn, prevents adverse perinatal outcomes. Delay in the provision of emergency obstetric care may put perinatal life in danger (1). In modern obstetrics, emergency cesarean section deliveries are part of emergency obstetric intervention, which can be offered for a variety of fetal and maternal indications (2). The decision to delivery interval (DDI) in a cesarean section (CS) refers to the timeline elapsing from the date and time of the decision to perform a cesarean delivery to the date and time of the delivery of the fetus. It is not similar to the decision to incision or decision to anesthesia time where the birth of the fetus is not achieved yet (3,4).

Regardless of the medico-legal controls in place to balance patient safety and service affordability, the need for emergency cesarean sections (ECS) has been increasing in low-resource setting (5). In sub-Saharan African (SSA) nations, the rate of CS accounts for 10–50% of all surgical procedures provided. One in seven cesarean sections in this region is attributed to a perinatal death (6). In Ethiopia, 13.0–14.7% of births in 2014 were delivered by cesarean section (7).

Recent studies raised confusion on the practicability of the “30-minute rule” for DDI and its beneficial influence on the favorable perinatal outcome (8). Even though it is a widely accepted criterion, evidence in support of a DDI within 30 minutes is scarce (9). The report underlines that 60–75 minutes may be more reasonable and justifiable for ECS in developing nations (10). Therefore, a DDI of less than 30 minutes is not an absolute threshold (3).

It has often been suggested that the speed of fetal delivery should be taken into account for assessing the state of fetal compromise and the predisposition to poor perinatal outcomes (11, 12). On the contrary, if the 30-minute DDI is good and 20 minutes is better, in previous literature, the outcome measures were found worse as time went below 30-minutes (13). The correlation between the performance of ECS and the subsequent outcome may determine the level of adherence to the 30-minute DDI (13). Evidence suggested that the need for neonatal intensive care unit (NICU) admission was more common in laboring mothers whose cesarean delivery lasted more than 30 minutes. The number neonates who had developed acidosis or asphyxia in this group increased (14).

Despite all the concerns about obtaining emergency obstetric care, data show that the relationship between DDI and the neonatal outcome is lacking (7). Ethiopia has endorsed strategies to avert the burden of poor perinatal outcomes through the provision of a skilled birth attendant during childbirth. Despite the interventions made to improve perinatal outcomes, there is a need to give attention to newborn interventions (15). Given the busy and often congested maternity units in Ethiopia, especially in the study area, it would be reasonable to examine the decision-making delivery interval and its relationship with perinatal outcomes. Therefore, this study aimed to assess the decision to delivery interval, perinatal outcome, and its associated factors in Gamo Zone hospitals, Southern Ethiopia.

## Methods and Materials

Study setting and design: An Institution-based cross-sectional study was conducted in Gamo Zone hospitals in southern Ethiopia from November 1, 2020, to January 30, 2021. Gamo Zones is one of the independent administrative zones in the southern nation, nationalities, and people regional state and is located in the south of Addis Ababa (the capital city of Ethiopia). Gamo zone has one general hospital (Arba Minch General Hospital) in which comprehensive emergency obstetric and perinatal care is available, and four district hospitals (Chencha primary hospital, Kamba primary hospital, Selamber primary hospital and Gerese primary hospital) all have emergency cesarean section service.

Populations

All women who underwent emergency cesarean section in Gamo Zone public hospitals were observed as the source population. All women who underwent emergency cesarean sections during the data collection period and who fulfilled the inclusion criteria were included in the study population.

**Eligibility criteria:** Women who underwent emergency cesarean sections for intrapartum care were included. Multiple pregnancies, IUGR, and congenital anomalies were excluded, as perinatal morbidity is known to be higher in this group and the babies cannot be treated independently.

**Sample size determination:** The sample size was calculated by using Epi Info version 7 menu Stat Calc programs for four potential determinants that were significant in recent studies with the consideration of the following assumptions: a confidence level of 95%, a power of 80, and exposure to an unexposed ratio of 1:1, which is taken from the previous study done in Tanzania (29) (by taking the factor of prolonged DDI of more than 75 minutes), and the largest sample size was 496. 10% of the total sample size was added to compensate for non-response rate, and the final sample size was 546.

**Sampling techniques and procedure:** The number of women who underwent emergency cesarean sections in three-month periods in Arba Minch General Hospital, Chencha Primary Hospital, Selamber Primary Hospital, Kemba Primary Hospital, and Gerese Primary Hospital was 283; 112; 87; 93; and 96, respectively. It was taken from last year during the same period of achievement. Proportional allocation method was used. A consecutive sampling technique was used until the required sample size was achieved.

Decision to delivery interval and perinatal outcome are dependent variable while the independent variables are Socio-demographic factors (Age, Marital status, Educational status, occupation, and Income), Obstetrics related factors (antenatal care, transfusion, parity, gestational age, PROM, birth weight, previous c/s scar, current pregnancy disease, labor complication, labor companionship, smoking during and after pregnancy, drinking alcohol during and after pregnancy, Trail of labor, Failed instrumental delivery, and Gestational age) and Institutional and professional factors (availability of emergency triage, additional operation table, operation room linked to labor ward, operation team readiness, expert for operation and neonatal resuscitation, and blood availability).

**Operational Definitions of terms**

**Decision to delivery interval**: It is the timeline between the date and time of decision to perform an emergency cesarean section to the date and time of delivery of the fetus/baby/. It was categorized into 15 minute intervals (0–15, 16–30, 31–45, 46–60, 61–75, and > 75) (3).

**Emergency cesarean section: Category I**: Immediate threat to the life of woman or fetus and Category II: Maternal or fetal compromise but not immediately life-threatening was studied in the context of DDI.

**Neonatal outcome**: It was assessed in terms of delivery of fresh stillbirth, Apgar score<7/10, Apgar score ≥7/10, and NICU admission. Clinical data were examined to ensure the exclusion of intrauterine fetal deaths from stillbirths. Neonatal outcomes are categorized as unfavorable (APGAR <7 at 5 min or death) and favorable (alive and APGAR ≥7 at 5 min).

**Data collection method, instrument, and procedure**: Data were collected using an interviewer-administered questionnaire and a structured observation guide. The questionnaire was prepared in English and adapted from different works of literature, considering the study objectives. The questionnaire consists of five parts; socio-demographic, maternal health conditions during and before pregnancy, intrapartum factors, perinatal outcome, and reason for delay.

The data collectors and supervisors were recruited based on their experience with similar tasks at facilities other than the selected health facility. The data collectors approached the laboring woman once the decision had been confirmed by the duty physician and the parturient had consented, the data collectors started the observation process until the delivery of the newborn. Within three to four hours after the operation, the data collector approached the patient again for an interview on socio-demographics and other variables. The data collectors used a unique numeric identifier to track the mother's card along with their newborn to extract additional data from the operation and anesthesia notes.

**Data quality assurance**: The questionnaire was pre-tested in 5% of the sampled women in Sawla general hospital. An amendment on consistency, coherence, and terminology was made. Besides, the data collectors had two days of training on how to fill the questionnaires, conduct observations, and extract data from the patient card, and confidentiality issues. During the phase of data collection, the data collectors had a thorough check on the completeness of the questionnaire.

The supervisors also had to cross-check for the accuracy and consistency of data at the end of each data collection day. The validity of the data collection tool was assessed through review and feedback from two independent clinicians, and questions were sent to an additional expert for further validation. An independent biostatistician and an epidemiologist were consulted to make sure of the correctness of the analysis and its corresponding interpretation.

Data analysis and interpretation

Epidata 4.3 version software was used to code, enter, and clean the data. Then, it was exported to SPSS version 25. Descriptive statistics were carried out and summarized by tables, frequencies, graphs, means, and standard deviation. Categorical variables were compared using cross-tabulation and Chi-square tests as appropriate.

An association between perinatal outcome and DDI was examined using binary logistic regression. The adjusted odds ratio and confidence interval were calculated to determine the strength of the association. A p-value less than 0.05 with a corresponding 95% CI was declared as a statistically significant level of significance.

**Ethical consideration**: Ethical clearance was obtained from the Institutional Ethical Review Board of Arba Minch University College of Medicine & Health Science (IRB/2020). An informed consent was taken from clients /legal guardians of each minor group before data collection.

## Results

**Socio-demographic characteristics of respondents**: Five hundred thirty-four women participated in the study with a 97.8% response rate. The mean age of the study participants was 25.82 years (SD 5.03 years). More than half (52.2%) of the participants were rural residents.

Regarding marital status, 499(93.4%) of the study participants were married and 151(28.3%) of women had attended higher education. Around half of the respondents (49.1%) were unemployed; the median household monthly income of the study participants was 3500 ETB with an IQR of 3500 ETB (Table 1).

**Table 1:** Socio-demographic characteristics of the study participants among women who underwent emergency cesarean section in Gamo Zone hospitals, southern Ethiopia, 2020

Variables		No (%)
	18–24	225(42.1)
Age	25–34	271(50.7)
	≥35	38(7.1)
Residency	Rural	279(52.2)
	Urban	255(47.8)
Marital status	Married	499(93.4)
	Single	26(4.9)
	Others*	9(1.7)
Educational	No formal education	113(21.2)
level	primary	137(25.7)
	Secondary	133(24.9)
	Above secondary	151(28.3)
Occupation	Housewife	262(49.1)
	Government	103(19.3)
	employee	
	Farmer	77(14.4)
	Student	58(10.9)
	Others^	34(6.4)
Women	Yes	451(84.5)
admitted to	No	83(15.5)
labor ward		
Family monthly	<3500	274(51.3)
income	≥3500	260(48.7)

* widowed, separated

^ Daily labour, private/NGO

**The obstetrical and medical conditions of the mothers**: Two hundred eighty-nine (54.5%) of the study participants were multiparous. Of multiparous women, 169(58.5%) of them had a vaginal route of delivery, and 120 (41.5%) of them were delivered abdominally or in operation. Five hundred nine (95.3%) women had antenatal follow-ups during this pregnancy. Of all 99(18.5%) of the women who were alcohol drinkers, 27(5.1%) of them were chewing khat, and 7(1.3%) of the women were cigarette smokers during the current pregnancy.

Four hundred eighty women (90%) were at term pregnancy as a result of their LNMP or early ultrasound finding. And 85(15.9%) of those women were induced to start their labor. and oxytocin was the most common method (8.4%) followed by misoprostol and oxytocin (4.5%) combination method.

Among the study participants, 77(14.4%) of the women suffered from different medical illnesses during the index pregnancy, of those medical illnesses, the dominant one was preeclampsia 27(5.1%) followed by anemia 13(2.4%). Also 10 (1.9%) of women were transfused with whole blood preoperatively, intraoperative, and postoperatively. Three hundred six (57.3%) of the women used contraceptive methods, and 179(33.5%) of the women used injectable types of modern contraceptives. Of the women who underwent emergency cesarean section, 37(6.9%) were fasting before the operation was done. The study findings showed that 231(43.3%) of them were in the active first stage of labor and were followed by partograph (Table 2).

**Table 2:** Obstetrics and medical characteristics among women who undergo emergency cesarean section in Gamo Zone hospitals, Southern Ethiopia, 2020, (n=534)

	Variables and Categories	Frequency	Percent
Parity	Primiparous	245	45.9
	Multiparous	289	54.1
Previous route of delivery	Vaginal	169	58.4
(n=289)	C/ Section	120	41.6
VBAC(n=120)	Yes	49	40.8
	No	71	59.2
Ante natal care during the current	Yes	509	95.3
pregnancy	No	25	4.7
Alcohol drinking during	Yes	99	18.5
pregnancy	No	435	81.5
Smoking cigarette during	Yes	7	1.3
pregnancy	No	527	98.7
Chewing khat during pregnancy	Yes	45	12.4
	No	318	87.6
GA based on LMP/US	preterm	10	1.9
	Term	480	89.9
	Post term	10	1.9
	Unknown	34	6.3
Induction of labor	Yes	85	15.9
	No	449	84.9
Methods of induction (n=85)	Oxytocin	45	52.94
	Misoprostol	8	9.41
	Aminiotomy	6	7.05
	Misoprostol & oxytocin	20	23.52
	Miso, oxy & amniotomy	6	7.05
Types anesthesia	General	17	3.2
	Regional	517	96.8
Stage of labor at time decision	Active first and second stage	231	43.3
	Latent & no labor	303	56.7
Mothers on fasting before the	Yes	37	6.9
operation	No	497	93.1
Routine laboratory test	Done	324	60.7
	Not done	210	39.3

GA- Gestational Age, LMP- Last Menstrual Period

**Figure 1:**
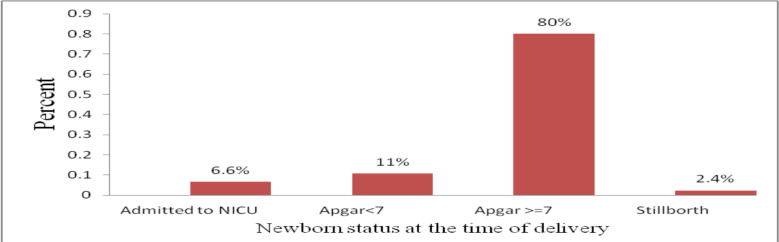
Neonatal status among women who undergo emergency cesarean section in Gamo Zone hospitals, Southern Ethiopia, 2020,(n=534)

**Perinatal outcome**: The majority 449 (84.1%) of the newborns who were delivered by emergency cesarean section had normal birth weight, regarding the fetal presentation 456(85.4%) of them were vertex presentations and more than half (54.5%) of the newborns had male sex. Four hundred twenty-seven (80%) of the newborns had a normal Apgar score at first and fifth minutes, 6.6% of the newborns were admitted to NICU, and thirteen (2.4%) of them were stillbirth. Of those stillbirths, 9(1.7%) of them were fresh and 4(0.7%) of them were macerated in appearance (Figure 1).

**Decision on delivery interval and perinatal outcomes**: The finding of this study on bivariate logistic regression analysis revealed that there was no statistically significant association between prolonged decision to delivery interval (*>*30 minutes) with both composite and individual adverse perinatal outcomes (like NICU admission, neonatal death before discharge, stillbirth, low Apgar score at 1st and 5th minute).

**Figure 2:**
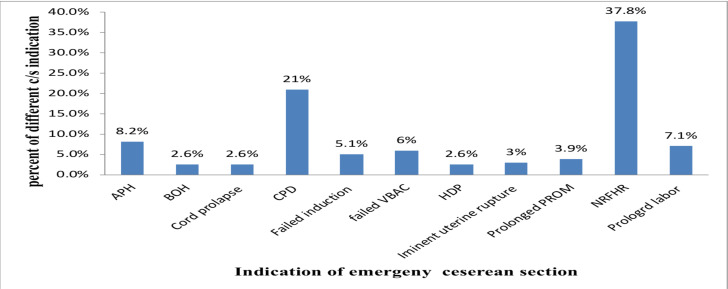
Indication of emergency cesarean section in Gamo Zone hospitals, Southern Ethiopia, 2020. (n=534).

**Indications for emergency cesarean section**: The principal indications for emergency C/S were non-reassuring fetal heart rate patterns (NRFHRP) 202 (37.8%) and Cephalo-pelvic disproportion (CPD) 112 (21%) followed by antepartum hemorrhage 44(8.3%) and prolonged labor 38(7.1%) (Figure 2).

**Facility and health care providers (HCPs) related characteristics**: Of all hospitals, only 2 of them had mini blood banks in their compound, and most of their operation rooms 4 were directly linked with the labor and delivery ward. Among those who responded, 493 (92.3%) had undergone emergency cesarean section and were triaged by obstetrics emergency triage. And also, for 441 (82.6%) of cases, functional additional operation tables were available while those women were prepared for operation, and for the majority of 479(89.7%) of cases, the operation team was ready to do the procedure without delay.

Regarding materials and drug availability, for 468 (87.7%) women, materials and drugs needed for emergency cesarean section were easily accessible in the hospitals during their preparation for the procedure. For four hundred eighty (89.9%) of women, health care providers give care to other clients besides women who are preparing for emergency cesarean section. Of those, 452 (94.1%) of the women, health care providers give priority to women who need an emergency cesarean section.

**Decision to delivery interval**: Of all study participants, only one hundred fourteen (21.3%) with 95% CI (18%, 25%) of parturients attained a decision-to-delivery interval within 30 minutes. Among women who had undergone emergency cesarean section more than half of the women 299(55.8%) were done during the night time and 44.2% of the procedure were done during the daytime.

**Factors associated with decision to delivery interval**: On bivariate analysis, admitted in labor, category of ECS, investigated for the routine lab test, presence of emergency triage ;, availability of additional operation table ; the readiness of the operation team ;, presence of materials and drugs needed for emergency cesarean delivery, duty time have a significant association with a short decision to delivery interval. To control the effects of the confounder, multivariable analysis was carried out. On multivariable analysis, the category of ECS, availability of additional operation table, presence of materials and drugs needed for emergency cesarean delivery, and duty time had a statistically significant association with a short decision to delivery interval.

Laboring women who are categorized as category one ECS (AOR=8.45, 95% CI, 4.66, 15.35) were eight times more likely to be accomplished decision to delivery interval ≤ 30 minutes than those women who are category two ECS. Parturient women who had an additional OR tables while they were preparing for EMCS had significantly greater odds of accomplishing their DDI within 30 minutes (AOR=3.31, 95% CI, 1.42, 7.70) than women who had no additional operation table.

Parturient women who had accessed materials and drugs needed for EMCS in the institution (AOR=4.08, 95% CI, 1.3, 12.62) were 4.08 times more likely to have completed DDI within 30 minutes than women who had not accessed materials and drugs in the institution. The odds of completing DDI within 30 minutes were three times (AOR=3.08, 95% CI, 1.04, 9.07) more among women who decided to undergo EMCS during the nighttime compared to women who decided to undergo EMCS during the daytime (Table 3).

**Table 3:** Bivariate and multivariable analysis of factors associated with decision to delivery interval in Gamo Zone hospitals, Southern Ethiopia, 2020, (n=534)

Variables	DDI completed within 30 minutes			Odd Ratio @ CI (95%)	
		Yes, N (%)	No,N(%)	COR	AOR
Women admitted in	Yes	89(19.7)	362(80.3)	0.57(0.33, 0.96)	0.71(0.37,1.35)
labor ward	No	25(30.1)	58(69.9)	1	1
Cesarean section	one	45(58.4)	32(41.6)	4.72(2.31, 9.62)	8.45(4.66, 15.35)*
category	Two	69(15.1)	388(84.9)	1	1
Routine investigations	Done	95(29.5)	229(70.7)	4.17(2.45, 7.07)	1.56(0.50, 4.86)
	Not	19(9)	191(91)	1	1
Presence of emergency	Yes	111(22,5)	382(77.5)	3.68(1.11, 12.15)	2.53(0.69, 9.27)
triage	No	3(7.3)	38(92.7)	1	1
Readiness of OR team	Yes	109(22.8)	370(77.2)	2.94(1.14, 5.57)	1.81(0.66, 4.95)
	No	5(9.1)	50(90,9)	1	1
Presence of materials	Yes	110(23.7)	355(76.3	5.03(1.79,14.14)	4.08(1.32, 12.62)*
and drug	No	4(5.8)	65(94.2)	1	1
Presence of additional	Yes	106(24)	335(76)	3.36(1.57, 7.16)	3.31(1.42,7.70)*
OR table	No	8(8.6)	85(91.4)	1	1
Duty time	Night	93(31.2)	205(68.8)	4.65(2.78, 7.74)	3,08(1.04, 9.07)*
	Day	21(8.9)	215(91.1)	1	1

* p-value <0.05

CI = Confidence Interval, COR = Crude Odds Ratio, AOR=Adjusted Odds Ratio

## Discussions

In our study, only one hundred fourteen (21.3%) of emergency cesarean deliveries were achieved with a recommended DDI of 30 minutes. The mean decision-to-delivery interval was 51 minutes, with a standard deviation of 17 minutes. This finding was consistent with the studies conducted at Oman(16), Bahr Dar(17), and the University of Gondar specialized hospital(18), which concluded that 23.8%, 20.3%, and 19.6% of parturient women who underwent emergency CS were born with the recommended DDI of 30 minutes, respectively. This similarity with Bahr Dar and Gondar hospitals may be due to the similarity in the difficulty of accessing materials and drugs for ECS in hospitals, less time elapsed, and practice and experience of health care providers or the operation team.

On the other hand, our result is higher than research conducted in the South Gondar Zone(19), Tanzania(20), Nigeria(21) and South Africa(22) which showed that only 17.5%, 12.3%, 5.7% and 5.2% of emergency CS was achieved by a DDI of less than 30 minutes, respectively. The variation might be explained by the time elapsed between studies and socio-demographic differences in the population in Tanzania, Nigeria, and South Africa.

This study showed that parturient women who had an additional OR table while preparing for EMCS were three times more likely to achieve their DDI within 30 minutes than women who had no additional operation table. This is supported by the study conducted in Oman revealed that women who had no additional OR table while they are preparing for EmCS were associated with a longer decision-to delivery interval(16). This might be attributed to the fact that if there is no additional OR table, it causes a prolonged decision to delivery interval secondary to the third delay.

This study found that a woman whose EmCS was performed with a ready OR team was four times more likely to have recommended DDIs than her counterpart. The findings of this study were in line with those of studies conducted in Oman, revealing that poor interdepartmental communication was deemed to be the main causative factor for DDI delays(16).

Based on our finding, the odds of achieving DDI within 30 minutes was three times higher among women who decided to undergo EMCS during the nighttime compared to women who decided to undergo EMCS during the daytime. A study conducted in Thailand revealed that when the CS decision was not made during working hours, the DDI was noticeably shorter than during working hours (23). It was also congruent with the study conducted in Uganda which stated that DDI was extended by CS performed in the daytime than those performed overnight(24). This may be explained by the operating table that could have been occupied by elective patients during working hours. This showed that the patient handling procedure can be simple during the nighttime; our findings also attributed due to that operation teams are more easily available during the night. However; studies conducted in Gondar and Singapore stated that EmCS performed during the daytime had a shorter decision-to delivery interval than those performed at night time(17, 25).

In the current study, women who had accessed materials and drugs needed for EMCS in the institution (AOR=4.08, 95% CI, 1.3, 12.62) were four times more likely to have completed their DDI within 30 minutes than women who had not accessed materials and drugs in the institution. This finding is congruent with the study done in Nigeria, lack of funds and non-provision of surgical material results in the delay of a decision to delivery interval(26).

A parturient woman whose EmCS was performed for category one EmCS indications was eight times more likely to have a shorter decision to delivery interval than those in category two. This is consistent with studies conducted in Bahr Dar, Ethiopia, Uganda, India, and Saudi Arabia (18, 27–29). This may be due to the feto-maternal conditions that need a prompt response from providers to save the life of the woman or fetus rather than maternal or fetal conditions, which are not life-threatening immediately.

The decision to delivery interval is a crucial period between the decision to undergo ECS and delivery of the newborn because averting adverse perinatal outcomes is critically time-dependent (7). Our study showed no statistically significant association between prolonged DDI (>30min) and perinatal admission, stillbirth, perinatal death until discharge, and less than seven 1st and 5th minute Apgar scores. The result is similar to study findings in Ethiopia Bahir Dar, South and North Gondar, Tanzania, and India (18, 27, 28, 30).

The finding of this study implied that DDI shorter than 30 minutes did not have an association with both individual perinatal outcomes and combined perinatal outcomes. This finding was supported by many study findings which showed that no significant association was revealed between a decision to delivery interval and perinatal outcome through emergency cesarean deliveries (23,24,31). Even though DDI has not shown a statistically significant impact on perinatal outcomes, it takes more than 30 minutes for most babies with negative events. The results have been supported by previous research indicating that higher DDI has not been statistically associated with bad perinatal outcomes (18, 32, 33). To conclude this study, Despite the importance of doing EmCS as the recommended interval for the improvements of a perinatal outcome as well as quality care for mother and newborn, a substantial number of women had not been achieved with the recommended DDIs (<30 minutes).

Women with category one EmCS, the availability of EmCS drugs and materials in the hospital, the readiness of the operation team, the presence of an additional operation table while preparing them for EmCS, and operating at night were more likely to be delivered within the recommended time (30 minutes). There was no statistically significant association between short decisions to delivery interval and both individual and composite adverse perinatal outcomes. Health facilities and health care providers need to be ready at any time to provide emergency care and have all the necessary materials on hand in the hospital, and every facility should have more tables in the operation theater.
